# Implementation of generative AI for the assessment and treatment of autism spectrum disorders: a scoping review

**DOI:** 10.3389/fpsyt.2025.1628216

**Published:** 2025-07-22

**Authors:** Jun-Seok Sohn, Eojin Lee, Jae-Jin Kim, Hyang-Kyeong Oh, Eunjoo Kim

**Affiliations:** ^1^ Department of Medicine, Yonsei University College of Medicine, Seoul, Republic of Korea; ^2^ Institute of Behavioral Sciences in Medicine, Yonsei University College of Medicine, Seoul, Republic of Korea; ^3^ Department of Psychiatry, Gangnam Severance Hospital, Yonsei University College of Medicine, Seoul, Republic of Korea

**Keywords:** autism spectrum disorder, generative artificial intelligence, large language model, machine learning, deep learning, mental health, natural language processing, scoping review

## Abstract

**Introduction:**

Autism spectrum disorder (ASD) is characterized by persistent deficits in social communication and restrictive, repetitive behaviors. Current diagnostic and intervention pathways rely heavily on clinician expertise, leading to delays and limited scalability. Generative artificial intelligence (GenAI) offers emerging opportunities for automatically assisting and personalizing ASD care, though technical and ethical concerns persist.

**Methods:**

We conducted systematic searches in Embase, PsycINFO, PubMed, Scopus, and Web of Science (January 2014 to February 2025). Two reviewers independently screened and extracted eligible studies reporting empirical applications of GenAI in ASD screening, diagnosis, or intervention. Data were charted across GenAI architectures, application domains, evaluation metrics, and validation strategies. Comparative performance against baseline methods was synthesized where available.

**Results:**

From 553 records, 10 studies met the inclusion criteria across three domains: (1) screening and diagnosis (e.g., transformer-based classifiers and GAN-based data augmentation), (2) assessment and intervention, (e.g., multimodal emotion recognition and feedback systems), and (3) caregiver education and support (e.g., LLM-based chatbots). While most studies reported potential performance improvements, they also highlighted limitations such as small sample sizes, data biases, limited validation, and model hallucinations. Comparative analyses were sparse and lacked standardized metrics.

**Discussion:**

This review (i) maps GenAI applications in ASD care, (ii) compares GenAI and traditional approaches, (iii) highlights methodological and ethical challenges, and (iv) proposes future research directions. Our findings underscore GenAI’s emerging potential in autism care and the prerequisites for its ethical, transparent, and clinically validated implementation.

**Systematic review registration:**

https://osf.io/4gsyj/, identifier DOI: 10.17605/OSF.IO/4GSYJ.

## 1 Introduction

### 1.1 Autism spectrum disorder and current care challenges

Autism Spectrum Disorder (ASD) is a neurodevelopmental condition characterized by persistent deficits in social communication and social interaction, along with restricted and repetitive patterns of behavior, causing lifelong challenges for affected individuals (1). About 1 in 31 children (3.2%) aged 8 years has been identified with ASD according to estimates from the Centers for Disease Control and Prevention (CDC)’s Autism and Developmental Disabilities Monitoring (ADDM) Network (2). In South Korea, a prevalence rate of 2.64% has been documented under conditions of active population-based screening (3). Given the high prevalence of ASD, its societal and economic impacts are profound. For instance, a 2015 study projected that the economic burden of ASD in the United States would reach USD 460.8 billion by 2025 (4). ASD is a growing societal concern, impacting individuals, families, and community systems worldwide.

However, despite increased awareness, the timely diagnosis and effective treatment of ASD continue to pose significant challenges in current clinical practice (5). Traditional ASD assessments rely primarily on parental reports or on manual observation by trained clinicians. This process is often time-consuming and resource-intensive, and its accuracy depends heavily on clinician availability and experience. These limitations frequently result in diagnostic delays and missed diagnoses, ultimately impeding early intervention efforts. Without timely and appropriate interventions, ASD places substantial pressure on healthcare systems, educational institutions, and social support services worldwide (6). Therefore, there is an urgent need to develop innovative tools and technologies capable of augmenting clinical expertise, enhancing the efficiency and accuracy of ASD screening and diagnosis, and facilitating personalized and timely interventions for affected individuals.

### 1.2 From artificial intelligence to generative AI in ASD care

To this end, AI represents a promising approach to addressing these critical challenges, offering opportunities to significantly enhance ASD care in terms of efficiency, accessibility, and scalability (7, 8). AI-driven tools and platforms not only have the potential to streamline diagnostic screenings and personalized interventions, but can also support caregivers, educators, and healthcare professionals by providing real-time guidance, monitoring therapy sessions, and delivering evidence-based recommendations (9). Such supportive technologies can substantially reduce provider workload, improve care consistency, and increase overall access to specialized services. Moreover, AI-powered analytical methods can identify meaningful patterns and trends across ASD care practices, informing policy decisions and optimizing the allocation of healthcare and educational resources at both institutional and systemic levels (10).

Although the application of AI technologies to ASD care remains in its early stages, with fewer than 30 empirical studies identified up to 2023 in a recent narrative review (11), the field is rapidly growing, driven by increasing interest and collaborative research efforts. For instance, the U.S. National Institutes of Health (NIH) awarded substantial funding to leading academic institutions in 2022 to enhance ASD understanding and develop innovative interventions (12). Among these initiatives, a major research grant was awarded to support the development of AI tools for detecting ASD in infancy and identifying brain-based biomarkers, highlighting a strong commitment at both institutional and governmental levels to integrating AI technologies into clinical practice (13). One notable example demonstrating the tangible impact of AI-driven approaches in clinical practice is the Cognoa ASD Diagnosis Aid, an FDA-authorized machine learning-based software designed to assist physicians in diagnosing ASD among children aged 18 months to 5 years exhibiting potential symptoms (14).

In recent years, GenAI, particularly large language models (LLMs) and multimodal models capable of jointly processing text and images, has emerged as a promising approach for enhancing mental health care, including ASD interventions (15–22). GenAI refers to computational models that can produce human-like outputs, such as text, speech, images, or videos, typically employing transformer-based neural network architectures trained on extensive datasets (15). The origin of GenAI can be viewed through multiple milestones. Technically, its foundation was laid in 2014 with the introduction of Generative Adversarial Networks (GANs), which enabled machines to synthesize new data. The introduction of the Transformer in 2017 marked a major architectural breakthrough, laying the foundation for modern LLMs. However, it was the public release of Chat Generative Pre-trained Transformer (ChatGPT) in November 2022 that marked the widespread adoption and societal impact of GenAI (17).

LLMs have garnered particular attention due to their remarkable ability to understand, generate, and reason about human language based on extensive pre-training on massive text corpora (15, 17). The potential of LLMs to deliver timely diagnostic suggestions and personalized therapeutic recommendations has spurred growing interest in their application to ASD, a condition that frequently involves interpreting subtle linguistic expressions and behavioral cues (9, 16, 23). Following the public release of ChatGPT in November 2022, research integrating GenAI, especially LLMs, into ASD care has significantly accelerated. Prominent LLMs including OpenAI’s GPT series, Google’s Gemini, and Meta’s LLaMA have demonstrated substantial capability in synthesizing medical knowledge and systematically analyzing unstructured clinical and behavioral data at scale (15, 24). LLMs are well-suited for ASD care due to their ability to process complex, context-rich inputs and draw on extensive knowledge. They can interpret conversational transcripts, clinical notes, and parental reports to identify autism-related indicators often missed by humans (20). As a result, LLMs are being explored for diverse applications, including the facilitation of more naturalistic dialogue, personalized therapy, and real-time behavioral monitoring (15).

**Figure 1:**
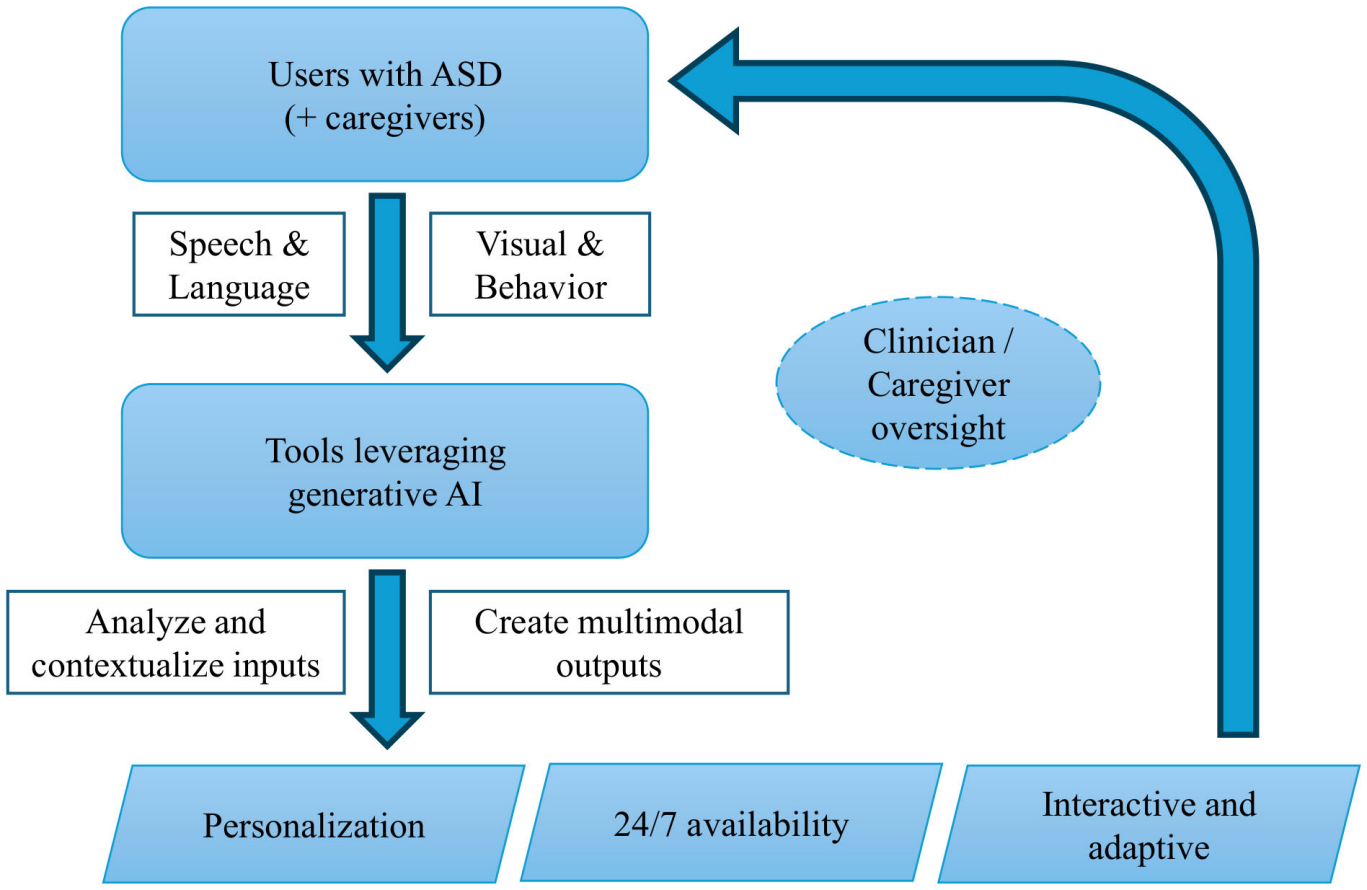
Interaction loop showing how generative AI models ingest multimodal data from autistic users, perform contextual analysis under clinician/caregiver oversight, and deliver adaptive feedback.

A major advantage of integrating GenAI into ASD interventions is their capacity for personalization, scalability, and consistent service delivery (20). Unlike standardized or “one-size-fits-all” approaches, GenAI-driven therapies can adapt dynamically to an individual’s interests, linguistic abilities, and emotional state—potentially enhancing engagement and sustained participation (18). Moreover, these interventions can be disseminated via accessible digital platforms (e.g., mobile apps, chatbots, social robots), thereby expanding support to families lacking specialized ASD resources (25). In contrast to human therapists—who may experience fatigue or variability in treatment delivery—AI systems provide consistent, centrally updatable interventions that incorporate the latest evidence-based practices. Nevertheless, most of these GenAI-based approaches remain in early prototype or experimental stages, highlighting the need for rigorous clinical validation to confirm their effectiveness, safety, and reliability (21, 22). **Figure 1** briefly illustrates interaction loop showing how generative AI models ingest multimodal data from autistic users, perform contextual analysis under clinician/caregiver oversight, and deliver adaptive feedback.

### 1.3 Research gaps and objective of the scoping review

Early studies have begun to explore the application of GenAI models in ASD care; however, the field remains nascent, with limited empirical synthesis. Several notable knowledge gaps emerge when reviewing the current landscape.

First, although various pilot studies and proof-of-concept systems exist, there has been no comprehensive synthesis of how GenAI technologies have been implemented across ASD screening, diagnosis, intervention, and caregiver support. A recent study noted a lack of systematic and comprehensive exploration in the field of methods based on LLMs (26). Second, the diversity and performance of GenAI model architectures in ASD contexts remain under-characterized, especially regarding multimodal data integration and real-world clinical validation (24).

To address these challenges, this scoping review aims to systematically map the application domains, implementation strategies, and outcomes of GenAI technologies in ASD care. Rather than offering a broad overview of AI in autism research, this study focuses specifically on empirically grounded applications of GenAI, including LLMs, GANs, and other transformer-based systems. In doing so, this review goes beyond the state-of-the-art by (i) synthesizing use cases and comparative performance data across studies (ii) characterizing model types and evaluation strategies; (iii) identifying methodological and ethical challenges; and (iv) outlining a research agenda for future development.

The review is guided by a set of structured research questions and objectives, presented in Section 2.1. Together, these elements offer a comprehensive view of how GenAI is being operationalized in ASD contexts and what is required to ensure its ethical, effective, and scalable integration into clinical practice.

The remainder of this manuscript is organized as follows. The *Methods* section outlines the scoping review framework, including search strategy, eligibility criteria, and data synthesis procedures. The *Results* section presents findings across application domains. The *Discussion* section interprets these results in light of methodological, clinical, and ethical considerations, and concludes with recommendations for future research.

## 2 Methods

The scoping review followed the Preferred Reporting Items for Systematic Reviews and Meta-Analyses Extension for Scoping Reviews (PRISMA-ScR) guideline (27). Our protocol was registered prospectively with the Open Science Framework on 20 February 2025 (https://osf.io/4gsyj/).

### 2.1 Research questions and objectives

The research questions and objectives defined to guide the search strategy and literature analysis were as follows:

Research Questions:

1. What are the main application domains of GenAI technologies in ASD care?
2. What GenAI methodologies are currently used in these ASD-related applications, and how are they implemented?
3. What benefits and limitations have been identified in existing studies applying GenAI for ASD?
4. What gaps remain in current research, and what future research directions are needed to advance GenAI applications in ASD care?

Research objectives:

1. To systematically identify and map the application domains of GenAI in ASD management, including screening, diagnosis, interventions, and caregiver support.
2. To characterize existing GenAI methodologies implemented in ASD-related applications.
3. To critically evaluate the benefits and limitations of current GenAI applications in ASD care.
4. To highlight key gaps in the existing research landscape and propose directions for future research.

### 2.2 Search strategy

To identify potentially relevant documents, the following databases were searched from 1 January 2014 (the introduction of GANs) up until 28 February 2025: PubMed, Embase, PsycINFO, Scopus, and Web of Science. The search strategies were drafted by a single reviewer [JSS] and further refined through team discussion. The final search results were exported into Excel, and duplicates were removed. Manual searches of reference lists in relevant reviews were conducted to supplement database results. The detailed search string for each database is provided in the **Supplementary Material**.

### 2.3 Eligibility criteria

The review process was guided by the PICO (Population, Intervention, Comparator, Outcome) framework, originally introduced by Richardson et al. (28). The PICO framework is widely utilized in evidence-based healthcare research to formulate structured, precise clinical questions and to facilitate systematic literature searches and analyses (29). The Cochrane Handbook also recommends the use of the PICO framework at various stages of developing research questions for systematic reviews (30).

The PICO framework defined for this scoping review was as follows:

1. Population (P): Children and adults diagnosed with ASD (any severity, any setting). Informal caregivers (parents, family members) and formal caregivers (clinicians, therapists) who directly use or supervise the AI tools can also be included.
2. Intervention (I): Application of GenAI tools (e.g. LLMs, text-to-speech, speech-to-text, image or video generators, etc.).
3. Comparison (C): Traditional interventions, usual care, or no active treatment/wait-list control, as applicable.
4. Outcome (O): Outcomes assessed included feasibility, effectiveness, or user experience of GenAI-based applications in ASD care.

Additional inclusion criteria applied in this review were as follows:

1. Studies reporting empirical findings on the use of GenAI in autism screening, diagnosis, or intervention.
2. Peer-reviewed research articles published between January 1, 2014, and February 28, 2025.
3. Studies available in full-text format and written in English.
4. Studies explicitly addressing GenAI-driven interventions involving social communication, behavioral management, caregiver education, or similar autism-relevant domains.

The corresponding exclusion criteria applied in this review were as follows:

1. Studies focusing solely on traditional machine learning or without GenAI components; Studies focusing predominantly on general lifestyle interventions, activities of daily living, self-care, independence skills.
2. Commentaries, editorials, reviews, conference abstracts, case reports, or non-peer-reviewed literature.
3. Studies not available as full-text articles or written in languages other than English.
4. Studies primarily examining technologies or methods such as neuroimaging, biomarker testing, genetic data analysis, biological samples, or neurostimulation.

### 2.4 Study selection and screening

Two independent reviewers [EJK and UJL] sequentially evaluated the titles and abstracts for relevance. Full-text screening was then conducted using the predefined eligibility criteria. Disagreements on study selection or data extraction were resolved by consensus or, when necessary, discussion with **a** third reviewer [JSS]. Inter-rater reliability was quantified with Cohen’s kappa value on a 10% random sample of records (31).

### 2.5 Bias mitigation

To minimize selection bias and ensure balanced coverage of perspectives, five safeguards were implemented:

1. Prospective protocol registration on the OSF and adherence to PRISMA-ScR guidelines.
2. Comprehensive database coverage spanning biomedical (PubMed, Embase), psychological (PsycINFO), and multidisciplinary (Scopus, Web of Science) domains.
3. Robust search strings combining controlled vocabulary (e.g. MeSH, Emtree) and free-text keywords for “autism spectrum disorder” and “generative AI”.
4. Independent dual screening by two reviewers, with arbitration by a third reviewer when required.
5. Assessment of inter-rater reliability using Cohen’s kappa value to verify the consistency of screening decisions.

### 2.6 Data charting

The following data-charting form was developed by reviewers to determine which variables to extract:

1. Study details (author, year, country, journal/conference, etc.)
2. The core architecture of GenAI model employed (LLMs, multimodal models, etc.)
3. Application type (screening, diagnosis, treatment, intervention, others etc.)
4. Data sources (clinical records, speech analysis, parent reports, social media, etc.)
5. Performance metrics (accuracy, F1-score, sensitivity, specificity, etc.)
6. Comparison with traditional models (if applicable)
7. Outcome measures (e.g., cognition, language, social function, emotion recognition, empathy, social interaction, repetitive behavior, anxiety regulation, etc.)

The two reviewers [EJK and UJL] independently charted data, discussed the results, and continuously updated the data charting form on Excel file in an iterative process.

### 2.7 Critical appraisal of individual sources of evidence

To appraise the methodological quality of the included studies, we use the mixed methods appraisal tool (MMAT), version 2018 (32). This tool allows the critical appraisal of most common types of study methodologies and designs: qualitative research, randomized controlled trials, non-randomized studies, quantitative descriptive studies, and mixed methods studies. The included articles were assessed by one reviewer [JSS] and verified by a second reviewer [EJK].

### 2.8 Data synthesis

We categorized each study into three broad groups: (1) screening and diagnosis, (2) assessment and intervention (3) others (e.g., caregiver education, medical support, and user experience). Findings were synthesized using a narrative approach. Key findings, advantages and limitations, and future research direction were highlighted.

## 3 Results

**Figure 2:**
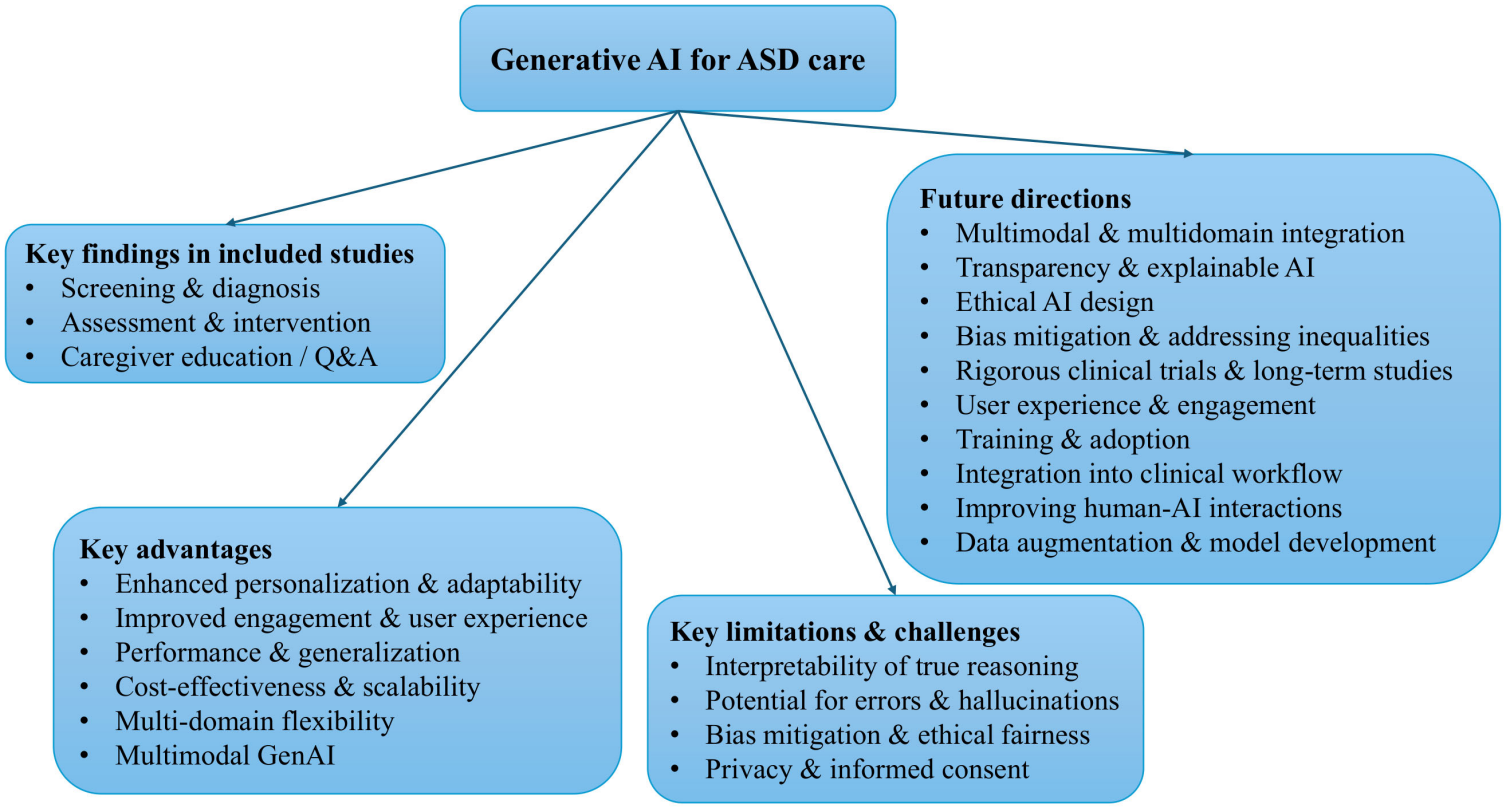
Taxonomy of GenAI research for ASD care. This hierarchical diagram maps the landscape of GenAI applications in ASD. The root node branches into four top-level categories: (1) key findings in the included studies, (2) key advantages, (3) key limitations and challenges, and (4) future research directions.

**Figure 2** shows how the current body of research distributes throughout the article.

### 3.1 Selection of sources of evidence

**Figure 3:**
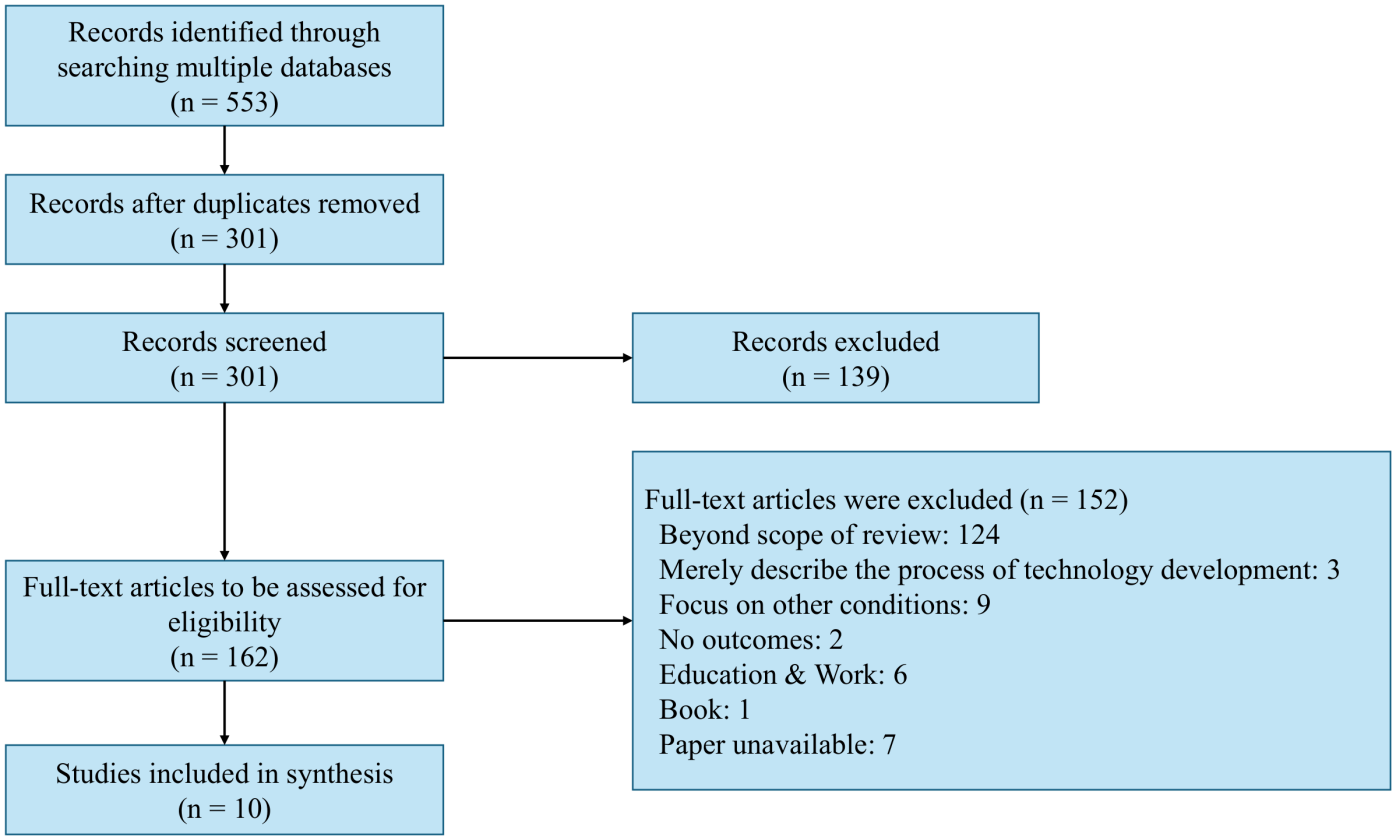
Flow diagram of study selection process.

A flow diagram illustrating the study selection process is presented in **Figure 3**.

The inter-rater reliability of screening was calculated as 0.79 for titles/abstracts and 0.86 for full texts, respectively. Cohen suggested that kappa values <0.20 indicate none to slight agreement, 0.21-0.40 fair, 0.41-0.60 moderate, 0.61-0.80 substantial, and ≥0.81 almost perfect agreement (31). The kappa values obtained in our review indicate substantial to almost perfect inter-rater agreement.

A total of 553 articles were identified from Embase (n = 95), PsycINFO (n = 10), PubMed (n = 85), Scopus (n = 278), and Web of Science (n = 85) databases. After removal of 252 duplicates, 301 records were eligible for further screening. Initial screening based on titles and abstracts led to the exclusion of 139 records, leaving 162 articles for full-text assessment. During the full-text review, 152 articles were further excluded for the following reasons: 124 were beyond the scope of this review; 3 merely described the progress of technology development; 9 focused on conditions other than autism spectrum disorder; 2 lacked clear outcomes; 6 were education and work; and 1 was a book. Additionally, 7 studies were excluded because their full texts could not be retrieved. Ultimately, 10 studies met all eligibility criteria and were included in the scoping review (33–42). Critical appraisal of included articles using MMAT checklist are provided in the **Supplementary Material**. A list of the included studies and the study characteristics are reported in **Table 1**. From the ten selected publications, the first authors were affiliated with institutions in the United States and China (n = 3 each), followed by India (n=2), Germany and Japan (n = 1 each).

**Table 1:** Characteristics of included studies.

Author (year)	Objective	Generative AI	Comparator	Subject	Outcome
Deng et al. (34) (2017)	Diagnosing autism spectrum condition based on speech patterns	GAN	Linear SVM; RBF-SVM; MLP (4 hidden layers)	Speech data from 34 monolingual children (AD: 11, PDD-NOS: 10, SLI:13)	GAN (layer 3) + SVM achieved the best test UAR (44.06%, +10.40% over linear SVM baseline)
He et al. (33) (2024)	Evaluating LLM chatbot effectiveness for autistic patients’ online queries	ChatGPT-4; ERNIE Bot 2.2.3 version	Physicians’ responses	A total of 239 autism-related online consultation queries from 100 patients	Physician responses were preferred (46.86%) over ChatGPT (34.87%) and ERNIE bot (18.27%), scoring higher in relevance, accuracy and usefulness; ChatGPT ranked highest in empathy
Koegel et al. (35) (2025)	Improving empathetic communication in autistic adolescents/adults	GPT-4-0613	Waitlist control	A total of 30 autistic participants (11–35 years old, mean age = 18.5 years); two groups (n = 15)	71% of the experimental group improved from their first to last 50 responses (avg. ~6.6 more correct responses); generalized to social interactions
Kurian et al. (36) (2024)	Emotion recognition in autistic children using combined facial and vocal expressions	Wasserstein GAN	Other various existing techniques	Videos of 75 children showing stereotypical behaviors (e.g., head-banging, stimming, spinning)	The proposed model outperformed other recognition classifiers, achieving an accuracy of 88.25%.
Lyu et al. (37) (2024)	AI and AR-based tablet game improving social-emotional learning in autistic children	GPT-3.5/4	Traditional slide-based learning	A total of 24 autistic children (mean age = 6.0 years, SD = 2.3) along with their caregivers	Intervention group improved emotion recognition quiz scores (mean +1.5/10); baseline group declined (-0.41)
Mukherjee et al. (38) (2023)	Detecting early ASD signs via parental behavioral narratives	Text-davinci-003 (GPT-3 Davinci family)	No specific comparator group	Dataset compiled from parents’ ASD-related dialogues on social networks/support groups	The proposed system used transformer/LLM models to classify ASD symptom sentiments (positive/negative)
She et al. (39) (2021)	Improving robot dialogue systems for autonomous interaction with autistic children	Generative CA that uses RNN-based Seq2Seq models	Canonical Seq2Seq model; GAN-based dialogue model	Healthy dataset: 4,398 scripts for training, 32 for validation, and 28 for training; ASD dataset: 374 scripts for training, 26 for validation, and 23 for training	The proposed model outperformed the standard Seq2Seq model and GAN-based dialogue models in autonomic and human evaluations
Tang et al. (40) (2024)	Supporting children with HFA in improving emotional recognition and expression skills	GPT-4; MidJourney niji-5	No specific comparator group	Six HFA children (5 boys and 1 girl), aged between 8 and 12 years old	Significant improvements from pre- to post-test were observed in (1) emotion recognition accuracy (2) emotion tokens indicating richer emotional expression, and (3) cosine distance suggesting more contextually appropriate vocabulary
Woolsey et al. (41) (2024)	Generating realistic textual examples of autistic behaviors to improve BERT-based model performance	GPT-3.5-Turbo; GPT-4	No specific comparator group	The dataset comprises expert-labeled CDC surveillance data from Arizona.	Clinician evaluation indicated 83% accuracy in randomly sampled LLM-generated behavioral examples. Data augmentation improved recall by 13% but reduced precision by 16%
Zhao et al. (42) (2025)	Automatically recognizing interaction styles in autistic children using audio-visual multimodal data annotated according to the FOS-II	GPT-4V (used as competing model)	Video recognition models: GPT-4V, CNN-based SlowFast Networks, and transformer-based Vision Transformer	A total of 216 home-recorded videos from 83 autistic children (mean age = 9.72 years, SD = 4.77), each lasting 5–15 minutes	The proposed model (AV-FOS) outperformed baselines (GPT-4V, SlowFast Networks, Vision Transformer) in accuracy (0.8590), F1-score (0.5936), and AUC (0.8868)

AD, Autism Disorder; AUC, Area Under the Curve; BERT, Bidirectional Encoder Representations from Transformers; CA, Conversational Agent; CNN, Convolutional Neural Network; FOS-II, Family Observation Schedule 2nd Edition; GAN, Generative Adversarial Network; HFA, High-Functioning Autism; LLM, Large Language Model; MLP, Multi-Layer Perceptron; PDD-NOS, Pervasive Developmental Disorder, Not Otherwise Specified; RBF, Radial Basis Function; RNN, Recurrent Neural Network; SD, Standard Deviation; Seq2Seq, Sequence-to-Sequence; SLI, Specific Language Impairment; SVM, Support Vector Machines; UAR, Unweighted Average Recall.

### 3.2 Key findings in included studies

**Table 2** presents the studies that compare the GenAI approach with baseline methods, along with the metrics and outcomes used for comparison.

**Table 2:** Head-to-head results for GenAI approach versus alternative baselines on autism-related tasks.

Study	Task	GenAI approach	Comparator(s)	Metric(s)	Δ (vs best comparator)
Deng et al. (58)	Speech-based diagnosis	GAN + SVM	Linear SVM, RBF-SVM, MLP	Unweighted average recall (UAR)	+3.09 pp (vs MLP)
Zhao et al. (42)	Interaction style (FOS-II) recognition	AV-FOS*	GPT-4V, SlowFast, ViT	Accuracy/F1 Score/AUC	+3.0 pp accuracy (vs SlowFast)/ +4.9 pp F1 Score (vs ViT)/ +4.2 pp AUC (vs ViT)
Woolsey et al. (41)	Data synthesization	GPT-3.5/4 generated data + BioBERT	BioBERT (no augmentation)	Recall/Precision	+13 pp recall/ -16 pp precision
Koegel et al. (35)	Empathy training	GPT-4-based chatbot	Wait-list control	Proportion of correct empathic responses	+35 pp improvement
Lyu et al. (37)	AR emotion-recognition training game	GPT-4 content	Traditional slides-based social-emotional lesson	Quiz score change (pre-post, 10-point)	+1.91 pts greater gain
He et al. (33)	Online caregiver Q&A	ChatGPT-4, ERNIE	Physicians	Evaluator preference/Relevance, Accuracy, Usefulness, Empathy (5-point Likert)	-12 pp preference/ +0.06 relevance, +0.07 accuracy, -0.14 usefulness, +0.51 empathy

Positive Δ values denote improvements of the GenAI approach over the strongest baseline for each metric (pp, percentage points; pts, raw points).

*Generative component limited to masked-autoencoder pre-training; inference is discriminative.

#### 3.2.1 Screening and diagnosis

LLMs and other GenAI approaches have demonstrated promising potential in enhancing ASD screening and diagnostic processes. This category encompasses studies utilizing GenAI models to support early ASD screening, assisting clinical decision-making, or derive meaningful insights from clinical datasets. Specifically, it includes diagnostic classifiers that use behavioral or linguistic features, intelligent rating systems to streamline or automate standard assessment tools, and predictive models that estimate ASD risk from diverse data sources. Additionally, GenAI can augment limited clinical samples by producing synthetic datasets or simulate diagnostic scenarios (43).

Mukherjee et al. (2023)’s study explores the application of AI models, specifically Bidirectional Encoder Representations from Transformers (BERT) and ChatGPT, in identifying early signs of ASD through parents’ narratives about their children’s behaviors (38). Texts were collected from social networks and ASD support communities, then labeled as positive (ASD-related) or negative. BERT, a transformer-based model, was fine-tuned for binary classification, achieving 83% accuracy with precision scores of 0.84 (negative) and 0.87 (positive), and F1-scores of 0.85 and 0.79 respectively. Although the study does not provide explicit numerical performance metrics for ChatGPT’s ‘text-davinci-003’ model, which was trained using reinforcement learning from human feedback, it reports that the model demonstrated consistent performance on new data. For positively labeled sentences, the system calculated cosine similarity with a curated library of ASD symptom statements to identify specific challenges, such as speech delay or poor eye contact, enabling more personalized intervention recommendations. This approach offers a scalable, non-invasive alternative to traditional diagnostic methods, particularly valuable in resource-limited settings. However, the authors acknowledge several limitations, including a small dataset size (although the article does not specify the exact number of examples, the reported evaluation metrics suggest that only about 80 sentences were used); data-collection procedures and class distribution, which limit reproducibility and transparency; reliance on subjective parent input; a lack of cultural diversity; and potential inaccuracies introduced by AI-generated outputs. Despite these challenges, the study demonstrates the potential of GenAI to enhance early ASD detection. Carefully curated datasets drawn from parents’ lived experiences are essential, as they may capture subtle behavioral cues overlooked by formal screening.

Deng et al. (2017)’s study explored the use of GANs for diagnosing ASD based on speech patterns (34). This method leverages machine learning to identify speech anomalies characteristic of ASD, such as echolalia, atypical prosody, and repetitive phrasing. However, it is widely recognized that the performance and reliability of such systems are constrained by the amount of available data for model training since annotated samples of autistic behavior or language can be scarce, and building large, representative ASD datasets is challenging and costly. To overcome this limitation, GANs were used to synthesize additional data to augment training sets. The authors evaluated the classifier’s performance on the database which includes over 6,380 utterances from 102 children across four categories (autistic disorder, pervasive developmental disorder-not otherwise specified, specific language impairment, and typically developing children) and compared the GAN-based method against three representative conventional models: a linear Support Vector Machine (SVM), an SVM with a Radial Basis Function (RBF) kernel, and a Multi-Layer Perceptron (MLP) with four hidden layers. Results show that the GAN-based approach achieved the best unweighted average recall (UAR) metrics of 44.06%, corresponding to a relative improvement of 10.4% over the Linear SVM baseline. The study concludes that GAN-based representation learning is a promising approach for ASD detection from speech, especially in contexts where data scarcity limits the performance of conventional models.

Another recent study demonstrates that modern LLMs can serve as “data generators” to produce realistic examples of autistic behaviors, which may be invaluable for data-hungry algorithms in diagnosis research. Woosley et al. (2024) investigates whether LLMs can generate realistic textual examples of autistic behaviors to enhance the performance of BERT-based neural networks (41). The study utilized a small corpus of real clinical observation data derived from CDC surveillance reports in the state of Arizona, comprising free-text entries labeled by trained experts. The dataset is highly imbalanced, with only 14.3% of sentences containing a diagnostic label, and the distribution of examples across autism symptom labels is similarly unequal. To address these limitations, the researchers presented a compelling proof-of-concept by prompting GPT-3.5-Turbo and GPT-4 to generate thousands of hypothetical child behaviors consistent with DSM-5 criteria for ASD. Specifically, they created 4,200 synthetic text snippets (e.g. “child repeats phrases from cartoons without understanding”) to represent ASD diagnostic features. A clinician review of a sample of the AI-generated cases found 83% were valid and correctly matched ASD symptoms. When the synthetic data were added to train a BioBERT model (44), the model’s recall improved by 13%. This indicates LLMs can help overcome data scarcity by providing additional examples of ASD manifestations, potentially boosting the sensitivity of screening tools. However, a precision drop in the augmented model (-16%) was noted, highlighting that synthetic data may introduce noise or irrelevant patterns.

Whereas previous studies have highlighted the strengths of GenAI models in ASD screening and diagnosis, recent study have shown that general-purpose generative models like GPT-4V perform worse than ASD-specific models in diagnostic tasks. Zhao et al. (2025) developed AV-FOS, a deep learning model for automatically recognizing interaction styles in ASD children using audio-visual data annotated with the Family Observation Schedule-Second Version (FOS-II) (42). The FOS-II is a validated tool for analyzing parent-child interactions but requires manual coding by trained observers, which is time-consuming and labor-intensive. AV-FOS was designed to address the limitations of manual annotation, while GPT-4V lacks domain-specific tuning. Compared to AV-FOS, GPT-4V (tested with Prompt Version 1 and 2), SlowFast Networks based on the Convolutional Neural Network (CNN) structure, and Vision Transformers based on the transformer structure showed lower accuracy, F1 score, and efficiency. Although the AV-FOS study excluded some rare annotations due to limited data, most omitted annotations had limited clinical significance. Overall, GPT-4V fell short in clinical applicability for behavioral assessment compared to the specialized AV-FOS model.

#### 3.2.2 Assessment and intervention

ASD affects communication, social cognition, and emotion recognition—domains where practice and feedback are crucial. AI technologies are therefore being designed to deliver scalable therapeutic assistance (10, 45, 46). The studies in this section examine GenAI tools that either assess the condition of individuals with autism or scaffold social-behavioral interventions.

Because emotional cues span facial expressions, body language, vocalizations, and even heart-rate signals, multimodal GenAI models can improve recognition accuracy—especially given autistic children’s challenges in expressing and interpreting emotions. Kurian et al. (2024) illustrate this with m_AutNet, a personalized framework that fuses facial and vocal data to recognize emotion in autistic children (36). A CNN-based visual embedder clusters images by similarity, while a transfer-learned CNN extracts audio embeddings; a Wasserstein-tuned GAN then aligns these domains for classification. The performance of the model was tested on the dataset comprising video recordings of 75 children who exhibit stereotypical behaviors such as head-banging, stimming, and spinning. In real-time emotion recognition tests, m_AutNet reached 88.25% accuracy, outperforming prior affect-recognition systems for the ASD children. Although the modest dataset limits generalizability, the results spotlight the value of personalized multimodal modeling in autism-focused emotion recognition.

Across these applications, the unifying aim is to strengthen autistic individuals’ social-behavioral skills through personalized and often interactive GenAI. Approaches range from LLM-based chatbots and socially assistive robots to virtual agents—tools increasingly aligned with digital-therapeutic trends in clinical practice (10, 25).

A clear example is EmoEden (40), which blends LLMs with text-to-image generators to help children with high-functioning autism (HFA) enhance their emotional recognition and expression skills. Parents input preferences so EmoEden can tailor story difficulty and dialogue flow. With personalized conversations and visuals that target common emotional challenges, children could practice identifying emotions, responding to others, and sharing their own feelings. Over 22 days, six HFA participants (8–12 years) showed marked gains in emotion recognition, emotional richness, and context-appropriate vocabulary. Observation records noted high completion rates and active engagement. Parents also described post-training gains, such as heightened empathy and willingness to express discomfort. GenAI allowed EmoEden to expand user input into richer expressions, though occasional unrealistic details emerged. Overall, EmoEden’s blend of personalized storytelling, supportive real-time feedback, and conversational models offers an accessible pathway for emotional learning, with broader implications for at-home training. Despite the promise of automated adaptation and reduced reliance on human labor, the authors stress that such AI cannot supplant therapists and must be carefully supervised to mitigate “hallucinations” or misdirected responses.

Koegel et al. (2025) similarly investigated Noora, a GPT-4-driven tool that delivers real-time feedback on empathetic responses (35). In a four-week randomized controlled trial (RCT) with 30 autistic adolescents and adults (11–35 years), each of the experimental and the waitlist control group completed 200 empathy trials. Each trial consisted of an empathy-inducing prompt, a participant-generated response, and immediate feedback. Findings revealed significant improvements in empathetic communication skills among participants, observed both within Noora practice sessions and generalized to social interactions; 71 percent of the participants in the experimental group showed an improving trend from their first 50 responses to their last 50 responses, with an average improvement of 13.2%. The experimental group showed a mean delta change score (pre- and post-intervention) of 37.67%, whereas the waitlist control group showed a mean improvement of 2.53%. Participants also reported increased confidence and high satisfaction levels regarding Noora. However, several limitations were noted. These included the absence of direct comparisons with traditional face-to-face empathy interventions, occasional discrepancies between Noora’s AI-generated evaluations and human raters’ judgments especially related to grammar or phrasing issues, a relatively small sample size, and the lack of long-term follow-up to assess sustained effectiveness. Despite these limitations, the study provides initial evidence supporting the potential of GenAI-based interventions in effectively enhancing empathetic conversational skills.

Traditional emotion-training methods often force children to stare directly at faces—an aversive task for many autistic learners. VR/AR environments can ease this discomfort (47). However, VR/AR interventions typically offer only pre-scripted scenarios with limited, multiple-choice responses that do not adapt to individual users. Integrating GenAI can bridge this gap between VR/AR practice and real-world application by introducing free-form dialogues that are tailored to each user, thereby assisting individuals with ASD in developing generalized social skills across diverse settings.

Building on this integration of immersive technologies and GenAI, a study by Lyu et al. (2024) introduced EMooly, a tablet-based game integrating GenAI and AR (37). This application aims to enhance social-emotional learning for autistic children through the active involvement of their caregivers. The system comprises five phases: 1) a customization phase, utilizing GPT-3.5/4 to generate personalized social stories for each child; 2) a comprehension phase, where the child read and understands these social stories, accompanied by the caregiver; 3) an observation and imitation phase, involving turn-taking exercises between the child and caregiver to practice mimicking facial expressions; 4) a recognition phase, which prompts children to engage in a dynamic AR activity to identify the target emotion from emotional expressions overlayed in the real-world environment; and 5) a reinforcement phase designed to consolidate the learned concepts with reflective questions. The researchers conducted a between-subjects controlled study with 24 autistic children (mean age: 6.0, 3 girls and 21 boys) and their caregivers, who participated in single-visit sessions at special education centers. The study compared EMooly to a traditional slide deck-based intervention method, serving as a baseline. Results indicated that EMooly significantly enhanced children’s emotion recognition skills and outperformed the baseline method in terms of usability and overall user experience. Specifically, children with EMooly showed larger improvements on quizzes assessing emotion recognition abilities (scored out of 10) from pre- to post-intervention, with an average increase of 1.5 points, whereas the baseline group exhibited a mean decrease of 0.41 points. In sum, GenAI is poised to make VR/AR social skills training more immersive and personalized, but ongoing evaluation will be needed to measure whether these AI enhancements using LLM translate to better real-world social outcomes.

Extending beyond virtual environments, GenAI is also being integrated into physical interaction platforms such as social robots. In particular, social assistive robots (SARs) have long been explored in ASD therapy as engaging and judgement-free interaction partners (48). These robots can generally be divided into two types: retrieval-based and generation-based. Retrieval based models select responses from a predefined repository, limiting their interactions to pre-recorded dialogues. However, when relevant conversational scenarios are absent from the database, autistic children may quickly lose patience or interest. In contrast, generation-based models leverage GenAI, allowing robots to create responses dynamically beyond predetermined scripts, resulting in more natural and flexible conversations. Recently, researchers have explored combining LLMs with social robots, where the LLM manages language interactions and the robot provides visual and interactive engagement, enhancing user experience (46).

One such implementation of this LLM-augmented social robotics approach is demonstrated in the study by She et al. (2021) (39). This study aimed to improve the conversational abilities of the humanoid robot NAO when interacting with autistic children by developing a deep learning-based dialogue model. Using sequence-to-sequence architecture enhanced with attention mechanisms and GloVe word embeddings, the researchers trained the model through transfer learning—initially on dialogues from typically developing children, then fine-tuned with dialogues from autistic children. This model was integrated into the NAO robot and evaluated against several baseline models, including a standard Seq2Seq model and a GAN-based conversational agent (GCA), with and without BERT embeddings. The proposed model outperformed all baselines across multiple automated metrics, including BLEU score (0.23 vs. 0.15 for Seq2Seq), Greedy Matching, Embedding Average, Vector Extrema, and Skip-Thought similarity. It also achieved better semantic coherence and word distribution similarity with real conversational data, as reflected in lower KL divergence and Earth Mover’s Distance. Human evaluations conducted by 12 autism-experienced raters confirmed these results, with the proposed model scoring highest for single utterance quality (3.05) and overall script quality (3.23), compared to GCA (2.82 and 2.87) and Seq2Seq (1.89 and 1.83). These findings demonstrate that the model substantially enhances the naturalness, contextual relevance, and appropriateness of robot-generated conversations for children with ASD.

Another relevant work involved integrating the Pepper robot with ChatGPT to facilitate natural, open-ended dialogues with autistic individuals in real time (49). This study presented two different scenarios to leverage the robot’s capabilities to enhance communication, social skill development, and problem-solving abilities: 1) an informal interaction scenario focused on building rapport within a relaxed, comfortable environment; and 2) a structured interaction mimicking a psychoeducational setting, in which the robot posed problems for the users to solve. Although promising, this study primarily served as a feasibility demonstration, highlighting possibilities rather than confirming effectiveness through comprehensive evaluation.

#### 3.2.3 Caregiver education and medical Q&A support

Given the vast amount of online discourse surrounding ASD, ranging from scientific findings to misinformation, parents often struggle to find reliable resources about ASD. In response to this challenge, an LLM-based assistant has recently been proposed to provide on-demand, accurate information and guidance tailored to caregivers’ questions about ASD.

A recent study by He et al. (2024) assessed the effectiveness of LLM chatbots in addressing ASD-related questions (33). The researchers collected a total of 239 consultation queries posted by 100 randomly selected autistic individuals or their families from a web-based medical consultation platform in China. The answers were newly generated using OpenAI’s ChatGPT-4 and Baidu’s ERNIE Bot, which were then compared to answers previously written by human physicians. A panel of three chief physicians conducted evaluations of each answer. Evaluators preferred physician responses over those from chatbots. Also, physician responses achieved higher Likert scores in relevance, accuracy, and usefulness. The only exception was empathy, in which ChatGPT surpassed physician responses. This suggests LLM chatbots showed the possibility to augment patient or caregiver psychoeducation with a more empathic tone, although they may need further fine-tuning to match enough precision.

## 4 Discussion

This scoping review found that since 2017 and especially in the last three years of 2022 to 2025, researchers have begun to harness advanced AI (GPT-3, GPT-4, and similar models) to tackle some long-standing challenges in ASD diagnosis, intervention, and caregiver support. Early studies introduced AI-driven assistive technologies, such as socially assistive robots and smart glasses to improve emotional/social behaviors and inclusion for autistic individuals (8, 34, 46, 48–50). These foundational efforts paved the way for recent breakthroughs after 2022, where generative multi-modal models are revolutionizing both diagnosis and interventions for ASD care (17, 51, 52). They are enhancing scalability and accessibility by delivering personalized and engaging interventions through digital platforms, particularly in under-resourced areas. Crucially, AI systems are now being compared against traditional ASD practices, and in many cases delivering superior accuracy, efficiency, and accessibility (11, 35, 50–52).

Generative AI technologies are beginning to significantly reshape the landscape of ASD care. In diagnostic contexts, transformer-based language models such as GPT can analyze textual or behavioral data, identifying subtle linguistic or behavioral markers indicative of ASD with promising accuracy. In intervention contexts, GenAI has facilitated more naturalistic and engaging social interactions via interactive digital agents such as conversational agents, VR avatars, and SARs. These AI-enabled tools show promise as supportive resources for autistic individuals, offering continuous availability, consistent interactions, and non-judgmental support. Early evidence, although limited in scope, indicates benefits including faster screenings, measurable improvements in communication and social skills, and high levels of user engagement and satisfaction (11).

One important advantage of GenAI systems lies in their ability to deliver highly personalized interventions. Unlike conventional, resource-intensive, or standardized approaches, GenAI systems can dynamically adapt interactions in real-time based on each individual’s unique learning profile, interests, and emotional state (18, 21, 22, 53). This capability is particularly relevant given the significant heterogeneity among autistic individuals. Furthermore, GenAI technologies offer scalable, cost-effective intervention delivery, potentially available 24/7, thus substantially reducing barriers to accessing specialized ASD services (18, 22).

Caregivers and families of autistic individuals may also benefit from GenAI-based support systems. For example, recent studies suggest that LLMs, such as ChatGPT, can provide caregivers with accurate, empathetic, and accessible autism-related information, complementing professional guidance (33). However, these models currently exhibit limitations, including insufficiently nuanced or actionable information, emphasizing the continued need for careful supervision and refinement (33).

Additionally, GenAI models hold potential to address data scarcity challenges prevalent in ASD research. Synthetic data generation approaches using generative models can produce realistic behavioral or linguistic datasets from textual descriptions or limited behavioral indicators, thereby supporting the development and training of robust machine learning-based diagnostic and intervention systems (41).

Despite these promising developments, it is important to recognize that most GenAI-based ASD applications remain at early proof-of-concept or prototype stages. The reviewed studies typically involve small sample sizes and relatively short-term evaluations, limiting the generalizability and robustness of current findings. Nevertheless, preliminary results are encouraging, demonstrating near-human-level performance in specific contexts. For instance, AI screening tools achieving diagnostic accuracy comparable to expert clinicians, or AI-based interventions yielding therapy-like improvements in social and communicative skills. At the same time, this rapid progress underscores the critical need for thoughtful implementation, rigorous clinical validation, ethical considerations, and alignment of AI-driven tools with established clinical standards and individualized patient needs (9, 53–55).

Based on the most frequently addressed themes in the reviewed literature, this discussion highlights the key advantages, identifies remaining challenges, and suggests future directions for research and clinical practice involving GenAI in ASD care. As a scoping review, the current synthesis aims to provide a comprehensive and critical perspective on emerging applications in this rapidly evolving field. Along with the discussions, **Table 3** summarizes ten high-priority research avenues identified in our scoping review and maps each to concrete methodological improvements, specific research questions, and near-term actions.

**Table 3:** Action-oriented roadmap for advancing GenAI research and deployment in ASD care.

#	Research priority	Methodological enhancements needed	Illustrative research question(s)	Actionable next steps
1	Multimodal & multidomain integration	• Architectures that fuse text, audio, vision, motion, etc. • Large, demographically balanced benchmark datasets	*How does adding synchronized eye-tracking and prosody signals change Gen-AI based symptom-severity predictions?*	- Build a public, cross-site multimodal warehouse - Publish a baseline fusion model with open code & error-analysis sheets
2	Transparency & explainable AI (XAI)	• Attention-map visualization for generative models • Counter-factual & concept-activation techniques adapted to ASD features	*Which linguistic cues drive an LLM’s recommendation of “speech-therapy” for a given child?*	- Release an open-source XAI toolkit for ASD-GenAI - Conduct clinician‐rated usefulness studies of generated explanations
3	Embedded ethical-AI interface	• Real-time algorithmic-risk audits • Participatory design protocols with autistic stakeholders	*Does an ethics-by-design workflow reduce deployment-stage incidents (e.g., hallucinated advice)?*	- Pilot interdisciplinary “GenAI-ethics rounds” during model development sprints - Share checklists/templates for embedded-ethics reporting
4	Bias mitigation & equity	• Performance stratification by gender, culture & socioeconomic status • Fairness-constrained loss functions	*Do accuracy gaps shrink after targeted data collection in under-served regions?*	- Publish subgroup metrics in ASD-GenAI paper - Launch data-donation drives in LMIC clinics
5	Rigorous clinical & longitudinal trials	• Multi-site RCTs powered for functional outcomes • Long-term follow-ups	*Are gains from GAI-mediated social-skills training maintained in the long-term follow-up period?*	- Register pre-specified multi-site, long-term RCT protocols on ClinicalTrials.gov
6	User experience & sustained engagement	• Needs-assessment surveys for clinicians/parents • Competency-based training modules	*Which chatbot persona features maximize 6-month adherence?*	- Co-design interfaces with autistic self-advocates and their caregivers - Publicly release anonymized engagement datasets
7	Professional & caregiver adoption	• Real-time algorithmic-risk audits • Participatory design protocols with autistic stakeholders	*Does a micro-credential course increase therapists’ GenAI self-efficacy?*	- Develop accredited online training aligned with evidence-based practice - Trial AI-generated session summaries in real clinics
8	Clinical-workflow integration	• Cost-effectiveness modelling • Implementation-science frameworks (CFIR, RE-AIM)	*What organizational barriers impede EMR-linked GenAI decision support in community clinics?*	- Map reimbursement pathways with insurers - Conduct pilot implementations with process-evaluation metrics
9	Optimizing human-AI interaction	• Adaptive dialogue & embodiment strategies tuned to neurodivergent communication styles	*Does robot gaze-aversion timing affect engagement among minimally verbal children?*	- Run A/B tests on interaction modalities (text vs. voice vs. robot) - Publish design guidelines for ASD-friendly prompt engineering
10	Data augmentation & ASD-specific model development	• Validity tests for synthetic behavioral/neuroimaging data • Custom architecture search for ASD tasks	*Can diffusion-generated social-scenario videos improve emotion-recognition classifiers?*	- Release benchmark synthetic datasets with provenance metadata - Host an open challenge on ASD-specific generative-data quality

### 4.1 Advantages and limitations of GenAI-based approaches in ASD care

#### 4.1.1 Key advantages

##### 4.1.1.1 Enhanced personalization and adaptability

LLMs exhibit notable strengths in processing unstructured data input, such as free-form text, conversational transcripts, and informal interactions (22, 56). This capability aligns particularly well with the narrative and interactive dimensions inherent in ASD diagnosis and intervention, contrasting with traditional machine learning approaches that typically require structured inputs, such as predefined sets of numerical features or standardized assessment scores (9). Furthermore, GenAI technologies offer the potential for highly personalized and adaptive interactions, a critical requirement given the substantial heterogeneity in individual profiles among autistic persons. Specifically, these AI-driven systems can dynamically tailor responses based on user input, progressively learn an individual’s unique communication style and interests, and autonomously generate personalized therapeutic contents, including therapy materials, therapeutic games, and customized social stories matched to each individual’s developmental level and personal preferences. Such capabilities not only significantly reduce preparation time for therapists but also ensure continual novelty and freshness of therapeutic content, thereby preventing rote memorization, and enhancing sustained attention.

##### 4.1.1.2 Improved engagement and user experience

GenAI’s ability to simulate natural, human-like conversations can enhance user engagement and motivation, particularly benefiting individuals with ASD who may find traditional social interactions challenging or stressful. By integrating multimodal communication channels, such as text, voice, and interactive visuals, GenAI tools can flexibly accommodate diverse preferences and sensory sensitivities common among autistic users, thereby providing a more engaging and personalized user experience. Empirical studies examining AI-based conversational agents in ASD care have reported positive user feedback, highlighting therapeutic rapport, interaction quality, and relevance of generated content as critical determinants of user satisfaction (50, 57). These findings indicate that GenAI technologies may meaningfully improve user experience and adherence to interventions, potentially leading to more sustained therapeutic outcomes.

##### 4.1.1.3 Performance and generalization

LLM-driven approaches offer significant advantages over traditional machine learning techniques for ASD-related applications, particularly regarding model performance and generalization across diverse contexts. Conventional ASD classification methods, such as support vector machines or early-stage deep learning networks, typically depend heavily on task-specific feature engineering and extensive training on limited, domain-specific datasets. In contrast, LLMs are pre-trained on vast, generalized text corpora, enabling them to effectively recognize and generalize autism-related linguistic and behavioral patterns with minimal or no task-specific training (e.g., zero-shot or few-shot learning) (18). This advantage is particularly impactful in ASD research, where autism-specific data are often scarce or challenging to collect, limiting the performance and robustness of traditional models.

For example, recent studies have demonstrated that generative LLMs can outperform specialized classifiers in accurately identifying subtle ASD-related markers, such as atypical language use or distinctive conversational patterns (58). Specifically, as highlighted in the results section of this scoping review, models such as ChatGPT have shown high diagnostic sensitivity in detecting linguistic and behavioral abnormalities associated with ASD. Consequently, the inherent ability of LLMs to leverage extensive prior knowledge and generalize effectively from limited autism-specific datasets makes them uniquely suited to addressing current challenges in ASD diagnosis and intervention, potentially enhancing diagnostic accuracy, sensitivity, and clinical applicability.

##### 4.1.1.4 Cost-effectiveness and scalability

LLMs offer significant potential for cost-effective and scalable ASD care, primarily due to their capacity for rapid processing of extensive datasets. For instance, LLMs can quickly analyze large volumes of patient data, including lengthy clinical interviews or extensive screening questionnaires, substantially reducing the time required for initial screening and diagnosis. By enabling timely identification of at-risk individuals, these models facilitate earlier intervention, which is critical in improving developmental outcomes.

Moreover, once adequately trained, GenAI models can be deployed at large scales with relatively low incremental costs, as they require minimal additional resources beyond computational infrastructure. Such scalability is especially important in regions where clinical resources are limited, or where waiting lists for specialized ASD evaluations and interventions are long. Additionally, AI-driven tools can operate continuously, providing on-demand screening, personalized support, or coaching services. This continuous availability significantly reduces barriers to early intervention and effectively supplements human clinical expertise, potentially leading to improved access and reduced disparities in ASD care.

##### 4.1.1.5 Multi-domain flexibility

Traditional machine learning models utilized in ASD research typically focus narrowly on specific tasks and modalities, such as computer vision algorithms dedicated solely to analyzing facial expressions or classifiers designed to evaluate particular standardized questionnaires (7). In contrast, LLMs exhibit exceptional flexibility and adaptability across diverse applications, significantly reducing the need to develop specialized algorithms for each separate task (18, 21, 24). This versatility represents a considerable advancement over earlier autism-focused AI tools. A single LLM framework can be effectively adapted to multiple roles and contexts simply by modifying input data or prompts (59). For example, the same GenAI system may screen social media posts for potential ASD-related traits (38), summarize a patient’s complex developmental history to assist clinical decision-making (54), or engage autistic individuals in tailored therapeutic dialogues (58). This multi-domain flexibility not only streamlines the development and implementation of AI tools but also facilitates their integration into diverse clinical, educational, and community-based settings, thereby amplifying their impact and reach.

##### 4.1.1.6 Multimodal GenAI

Early autism research has consistently shown that combining multiple data streams—speech, eye-tracking traces, video-recorded behaviors, physiological signals, and even genetic or neuro-imaging data—yields more accurate detection and richer phenotypic characterization than single-modality approaches (17, 51, 60–65).

As richer corpora are emerging—datasets that co-register video, gaze trajectories, autonomic measures, and clinician annotations alongside dialogue (16)—multimodal GenAI models have the potential to extend these gains from the research lab into practice. For example, m_AutNet, which jointly analyzes facial expressions and vocal cues, can infer emotional states more reliably, de-escalate stressful episodes, and deliver personalized emotion-recognition (36).

Beyond improved inference, multimodal GenAI offers two additional advantages. First, because the models accept prompts that intermix text, images, audio, and video, they can return responses in any of those formats: visual explanations for clinicians, synthetic social scenarios for therapy apps, or spoken feedback for caregivers (37). Second, their capacity to synthesize realistic multimodal samples can both augment sparse ASD datasets (58) and generate illustrative visuals that enrich digital intervention tools (40).

Although the field remains in its infancy, these capabilities position multimodal GenAI to catalyze the next wave of sensitive, context-aware, and truly personalized ASD assessment and care.

#### 4.1.2 Key limitations and challenges

Despite the aforementioned optimism surrounding the application of GenAI in ASD care, current research highlights several critical gaps and challenges that warrant careful attention in future work.

##### 4.1.2.1 Interpretability of true reasoning

One significant limitation of GenAI models, particularly LLMs, is their inherent lack of interpretability compared to simpler, more transparent machine learning approaches such as decision trees or linear classifiers. Earlier AI techniques could typically highlight specific input features (e.g., frequency of eye contact, distinctive speech patterns) that directly influenced classification decisions. In contrast, transformer-based neural networks, including LLMs, do not inherently provide intuitive explanations for their decisions. For instance, while an LLM might flag a child’s language sample as suggestive of ASD, it generally does not explicitly identify particular phrases, linguistic errors, or behavioral cues that contributed to that determination (21, 22).

To address this interpretability limitation, various methods, such as attention visualization, saliency mapping, and prompt-based explanation techniques, have been developed to enhance the transparency and interpretability of LLM reasoning processes in both academic research and clinical practice (15). However, these generated explanations may not necessarily reflect the model’s true computational reasoning. Instead, they represent plausible textual outputs generated by the model, lacking direct insight into the actual decision-making mechanisms. Consequently, although LLM-generated explanations can improve the perceived transparency of AI decisions by presenting results in accessible human language, the underlying decision-making processes remain largely opaque, similar to those of previous complex machine learning models (15, 22, 66).

Given this challenge, ensuring that LLM-based decisions are genuinely grounded in valid clinical evidence and systematically developing robust methods to verify AI reasoning processes represent crucial areas for further research (18, 22). Enhanced interpretability and transparency will be essential for building clinician trust, facilitating regulatory compliance, and ensuring responsible integration of GenAI into ASD care.

##### 4.1.2.2 Potential for errors and hallucinations

A second important challenge associated with LLMs is their propensity to occasionally generate incorrect, misleading, or entirely fabricated information, commonly referred to as “hallucinations” (15, 67). In the context of ASD diagnosis and intervention, this issue is particularly consequential. For example, an inadequately monitored AI system might incorrectly classify a neurotypical individual as autistic, or vice versa, especially when presented with ambiguous, noisy, or out-of-distribution input data (23, 52). Such errors pose significant risks in clinical and therapeutic settings, potentially leading to inappropriate clinical decisions, delayed interventions, or unintended harm to patients.

Thus, ensuring that GenAI systems consistently provide accurate, evidence-based responses is an ethical and clinical imperative. Current generative models, however, do not intrinsically guarantee factual correctness, and their output must therefore be rigorously validated and monitored. Effective content moderation strategies and validation mechanisms are necessary to promptly identify and correct inaccurate or misleading model outputs (15).

In this scoping review, although the reviewed studies indicate that ChatGPT and similar LLMs typically provide clear, accurate, and clinically-relevant responses to ASD-related queries, the risk of hallucination or misinformation remains a significant concern (33, 67). While ChatGPT has demonstrated diagnostic accuracy comparable to human clinicians in certain cases, the presence of even occasional inaccurate or misleading content underscores the critical need for cautious deployment, expert oversight, and continuous validation, particularly when implementing LLMs in sensitive medical contexts such as ASD diagnosis, intervention, and caregiver education (52).

##### 4.1.2.3 Bias mitigation and ethical fairness

Applying GenAI models for ASD care offer the potential to mitigate longstanding disparities in care, particularly across socioeconomically and geographically diverse populations (68). However, ensuring fairness and minimizing bias must underpin their development and deployment. Training datasets that insufficiently represent the full spectrum of ASD phenotypes—across age, gender, language, cultural background, and socioeconomic status—risk producing models whose predictions and recommendations lack validity for underrepresented groups (50). Currently, the global landscape of AI research and application in healthcare is characterized by marked inequalities, with most research originating from institutions within high-income countries (69, 70). This aligns with the finding in our scoping review, with no studies originating from low-income countries, and the majority were conducted in high-income settings, underscoring a geographic and demographic concentration of the evidence base. When AI systems trained predominantly on data from high-income countries are applied in low- and middle-income contexts, differences in healthcare infrastructure, patient demographics, and cultural nuances can severely limit accuracy and practical applicability, potentially exacerbating existing health inequities (71, 72).

Ensuring fairness and minimizing bias represent crucial ethical considerations when deploying GenAI models in ASD care. If training datasets do not adequately capture the diversity of the broader ASD population, for example, if they disproportionately represent specific age groups, gender identities, linguistic characteristics, or cultural presentations, the resulting AI models may generate predictions or recommendations that lack validity for underrepresented groups. Indeed, many existing studies rely heavily on English-language sources or samples drawn from narrow demographic distributions, potentially embedding algorithmic biases that reflect broader societal inequities (50).

In practical terms, an AI system trained without sufficient exposure to diverse linguistic, cultural, or developmental manifestations of ASD could systematically underperform for certain subgroups. Such biases may inadvertently reinforce or exacerbate existing disparities in ASD diagnosis and intervention access, including the historical underdiagnosis among minority populations. Therefore, ethical AI development must proactively incorporate thorough bias assessments, fairness evaluations, and systematic bias mitigation strategies. Designing GenAI systems with careful attention to inclusivity and equitable representation will be essential to prevent the inadvertent replication or amplification of historical biases, thereby ensuring robust and fair performance across diverse demographic subgroups (15, 73).

##### 4.1.2.4 Privacy and informed consent

The use of GenAI technologies in healthcare contexts introduces significant ethical challenges surrounding data privacy and informed consent, particularly due to the sensitive nature of developmental and behavioral health information (18). In this regard, chatbots powered by GenAI are especially concerning, as they have the potential to elicit sensitive personal information from users, often without their conscious awareness (74). This issue becomes even more critical in the context of ASD research and intervention, where data types such as video recordings, therapy session transcripts, clinical notes, and detailed developmental histories are commonly used.

Given the sensitive and personal nature of such data, robust guidelines, comprehensive ethical oversight, and transparent data-management practices are imperative. Adherence to established data privacy regulations, such as the Health Insurance Portability and Accountability Act (HIPAA) must be strictly maintained (73). Furthermore, clear and informed consent processes are essential to ensure that data subjects and their caregivers or guardians fully understand how their personal information will be used, stored, and managed within AI-driven systems.

Especially, most ASD AI tools will be used with children, raising special consent and autonomy concerns. Children may not fully understand how AI works and might take its prompts too literally, making age-appropriate and transparent interactions essential. Parents and clinicians must supervise and approve usage, similar to traditional therapies. Since GenAI can behave unpredictably, guardians must stay vigilant. Importantly, children should also have the right to refuse interaction if they feel uncomfortable, highlighting the need to balance AI benefits with respect for the child’s autonomy. In sum, establishing and rigorously enforcing standards for data privacy and informed consent is a non-negotiable aspect of ethically responsible AI deployment in ASD care.

### 4.2 Future directions

Despite the identified limitations, current research suggests that many existing challenges in applying GenAI to ASD care are addressable through targeted development efforts. For example, integrating multimodal data can mitigate the limitations associated with purely language-based models (16), and fine-tuning LLMs on autism-specific datasets or coupling them with structured, rule-based frameworks can significantly reduce errors and enhance accuracy. Thus, each limitation highlights specific research directions necessary for refining AI’s utility in ASD diagnosis and intervention.

Nevertheless, current findings also highlight several critical gaps and opportunities for future investigation. To fully realize AI’s potential in ASD care, subsequent research must prioritize improving transparency, robustness, interpretability, and rigorous real-world validation. Based on insights from our scoping review, we propose key research directions as follows.

#### 4.2.1 Multimodal and multidomain integration

While language is a crucial component of ASD evaluation and intervention, an ideal GenAI system should combine cues from tone of voice, facial expressions, eye gaze patterns, motion data, and even biological markers to form a complete picture. Developing such multimodal GenAI systems capable of seamlessly combining linguistic, visual, behavioral, and physiological data is an essential future direction, and recent work has started moving in this direction. For instance, advanced AI models could simultaneously analyze a child’s speech transcripts, social interaction videos, and wearable sensor measurements to generate individualized risk assessments or therapeutic recommendations (17, 50, 66). Achieving this goal necessitates large-scale, diverse datasets and innovative model architectures capable of effectively processing and integrating heterogeneous data streams (16).

In particular, ensuring data diversity is critical; future models must be trained on representative datasets encompassing various ages, cultural contexts, and functioning levels (67). Expanding multimodal databases and establishing shared data repositories accessible to the international research community represent concrete steps forward. Global collaboration and standardization of data collection methods and formats will help overcome current limitations related to small sample sizes and data scarcity (21, 75, 76).

Additionally, integrating AI tools across multiple domains through hybrid or ensemble approaches could significantly enhance diagnostic and therapeutic capabilities. For example, combining an LLM specializing in language processing with computer vision models proficient in emotion recognition can yield more accurate, robust, and generalizable systems (49, 52). Prior work in other neurodevelopmental contexts, such as comprehensive gait analysis in pediatric cerebral palsy, has demonstrated the value of leveraging multimodal data and deep learning to capture complex, clinically relevant patterns (77). Ultimately, multimodal GenAI has the potential to simulate multidisciplinary evaluations, synthesizing diverse information similarly to clinical expert teams, thereby enabling nuanced diagnostics, personalized interventions, and real-time adaptive support for individuals with ASD based on a wide range of real-time inputs.

#### 4.2.2 Transparency and explainable AI

As previously discussed, the limited interpretability of GenAI models presents a significant barrier to their adoption in clinical ASD care. Therefore, enhancing transparency and explainability of AI systems is a critical future research direction. Specifically, developing Explainable AI (XAI) methods tailored for generative models in ASD diagnosis and intervention could significantly improve clinician and family trust. Ideally, such models would not only provide diagnostic or therapeutic recommendations but also clearly highlight the underlying rationale, such as specific behavioral cues or language features, that informed their decisions (66).

While preliminary efforts in interpretable AI for ASD have begun, applying these techniques to large generative models remains challenging (66). Achieving progress in explainability for ASD-focused GenAI will require adapting broader XAI methodologies to the unique developmental and behavioral complexities characteristic of ASD. Future research should explore advanced explainability methods, including attention visualization which identifies the specific input elements influencing model outputs, and counterfactual explanations, demonstrating how different inputs could alter the model’s predictions (21). Additionally, integrating domain-specific clinical knowledge directly into model architectures could enhance interpretability without compromising performance (78).

#### 4.2.3 Ethical AI design

GenAI amplifies long-standing ethical challenges in autism research—fairness, privacy and informed consent—while introducing novel threats such as hallucinated content, bias amplification and cloud-based data-security vulnerabilities (76). To manage these risks, we advocate an embedded-ethics interface that couples mental-health practice with computing.

**Figure 4:**
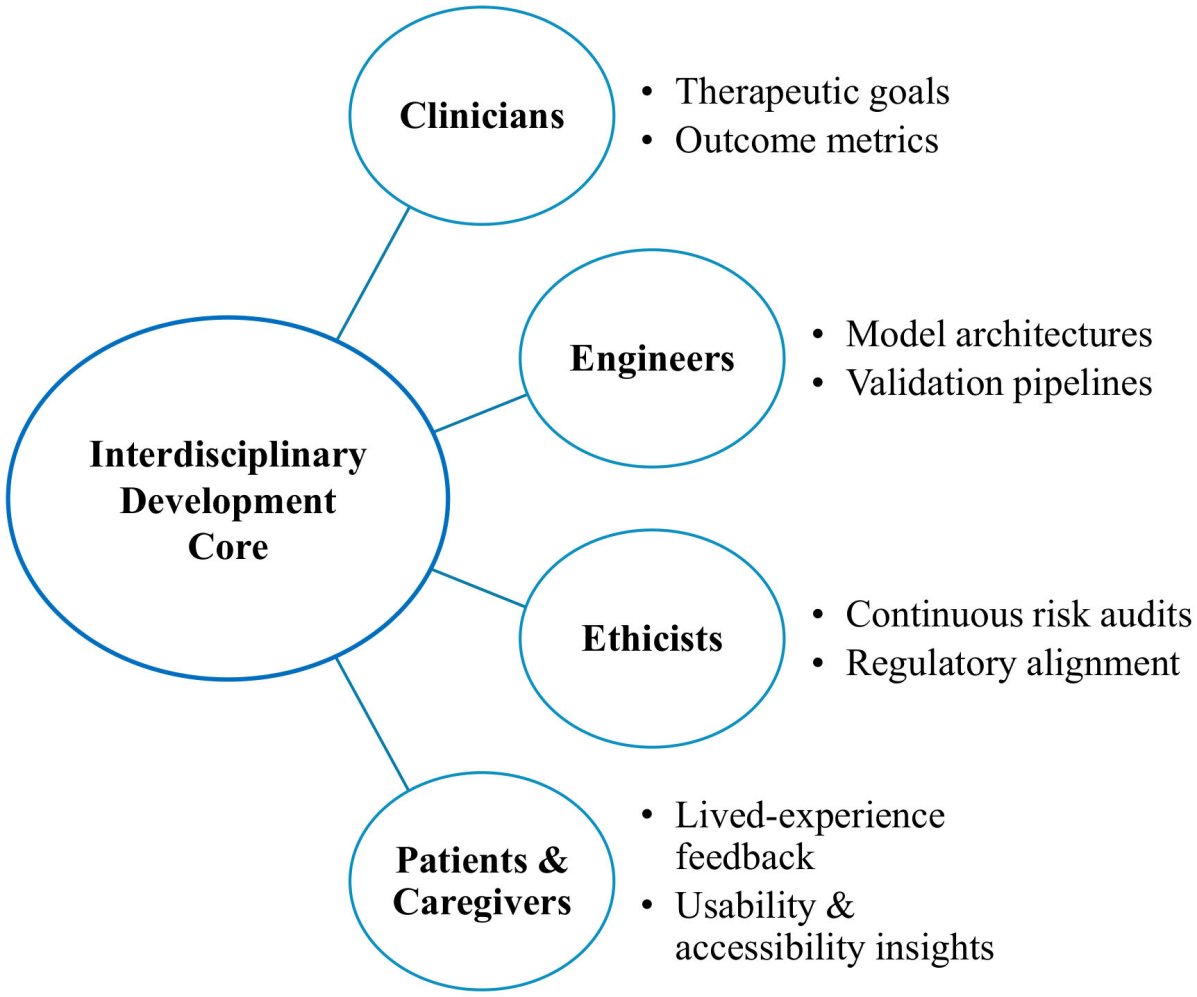
Quadripartite embedded-ethics interface for ASD-focused GenAI. Continuous collaboration among clinicians, engineers, ethicists and autistic stakeholders enables bias mitigation, real-time risk auditing and patient-centered design.

**Figure 4** illustrates a quadripartite interface in which (i) clinicians define therapeutic goals and outcome metrics, (ii) engineers translate these requirements into model architectures and validation pipelines, (iii) clinical ethicists perform real-time algorithmic-risk audits and regulatory alignment, and (iv) autistic patients and caregivers contribute lived-experience feedback. This continuous, bidirectional collaboration is intended to accelerate innovation while safeguarding patient safety and social equity.

Complementary scholarship substantiates the value of such an interface. McLennan et al. (2022) propose an embedded-ethics model in which ethicists join AI teams “from the workbench,” participate in sprint meetings and co-author methodological papers, thereby operationalizing real-time ethical scrutiny in medical-AI development (79). Cartolovni et al. (2022) map ethical, legal and social issues across algorithm, physician, patient and organizational layers and recommend an “ethics-by-design” workflow, conceptually aligned with our proposed interface (69). These precedents underscore the feasibility and necessity of embedding ethics expertise directly within ASD-GenAI development teams.

#### 4.2.4 Bias mitigation and addressing inequalities

While GenAI technologies have the potential to address shortages in healthcare professionals and reduce labor-related costs, such benefits must not come at the expense of exploiting vulnerable populations or exacerbating inequalities (80). Ensuring that GenAI serves as an equitable tool in ASD care requires verifying that training data accurately reflect the realities of target communities and that models are fine-tuned for specific cultural, linguistic and socioeconomic contexts (81). Ethically robust AI development for ASD must therefore incorporate comprehensive bias assessments and fairness evaluations, proactively include diverse linguistic and cultural representations, and pursue dataset diversification strategies—such as targeted data collection in underserved regions—to ensure equitable performance and avoid amplifying historical disparities (15, 73). Comprehensive international guidelines are therefore needed to address the distinct challenges of deploying these technologies in low- and middle- income countries (LMICs) and other resource-constrained environments (82).

Future work should quantify model performance across gender, ethnicity, culture and socioeconomic status in both diagnostic and interventional tasks. Where disparities emerge, corrective measures—dataset diversification, algorithmic debiasing or fairness-constrained architectures—must be implemented and transparently reported (9, 18, 83). In parallel, participatory co-design sessions with autistic self-advocates from diverse backgrounds can surface implicit assumptions, improve usability and foster trust (78). Ultimately, robust bias-mitigation pipelines and context-specific fine-tuning will be pivotal to realizing the promise of GenAI without accentuating existing inequities in ASD diagnosis and intervention.

#### 4.2.5 Rigorous clinical trials and long-term studies

Many GenAI-based tools for ASD (e.g., chatbots, robots, virtual reality programs, and mobile apps) have primarily undergone only preliminary pilot studies or evaluations in controlled or simulated environments. Before broader adoption, these interventions must demonstrate reliability, efficacy, and safety through rigorous clinical trials and secure appropriate regulatory approvals. Therefore, future studies should involve larger-scale RCTs evaluating meaningful clinical outcomes, including social functioning, adaptive behavior, and academic achievement, and directly comparing AI-enhanced interventions against standard care models (9, 18). Additionally, research must carefully assess possible adverse effects, such as increased screen dependency or unintended behavioral changes outside therapeutic contexts. Longitudinal studies are particularly crucial in ASD research, given the dynamic and evolving nature of developmental trajectories (9, 11). Long-term investigations can reveal whether improvements from AI interventions are sustained, generalized beyond training contexts, and positively influence life outcomes such as independence or employment in adulthood.

#### 4.2.6 User experience and engagement

Evaluating user perceptions and engagement is essential for successfully implementing AI interventions in real-world ASD care settings. Existing research indicates that autistic individuals often respond positively to AI-based tools, appreciating their predictability and nonjudgmental interaction style. However, future research should systematically address ongoing user engagement, adherence, and potential dropout rates, identifying factors influencing sustained use (25). Understanding long-term user experiences from the perspectives of patients, families, and clinicians will inform the development of AI tools that are engaging, practical, and effective beyond controlled research settings.

#### 4.2.7 Training and adoption by professionals and caregivers

GenAI technologies can potentially support not only direct ASD interventions but also training of caregivers and professionals. Successful adoption of these technologies will require targeted training and capacity-building among therapists, special educators, pediatricians, and caregivers to enhance their confidence and competence in utilizing AI effectively. While some clinicians may initially fear displacement by AI, a more realistic and beneficial scenario positions AI as assistive technology complementing human expertise (9). For example, LLMs could generate preliminary clinical reports from session notes, increasing efficiency (19, 44, 54); robotic assistants could handle repetitive therapeutic tasks, allowing professionals to focus on nuanced clinical decisions (11, 39, 45, 48, 57); or virtual patient simulations powered by GenAI could provide realistic training scenarios for novice practitioners (37). Clearly defining the complementary roles of AI and human professionals and providing adequate training and support will be critical steps toward successful integration into clinical ASD practices

#### 4.2.8 Integration into clinical workflow

Currently, most AI-based assistive technologies for ASD remain at the research stage, developed primarily within laboratories and not yet widely implemented into clinical practice (50). To facilitate clinical adoption, critical issues such as regulatory approval processes, insurance reimbursement policies, and clear evidence of cost-effectiveness must be addressed (15). Additionally, successful integration of GenAI tools into clinical environments poses pragmatic challenges. Practitioners will need training to use new technologies effectively, adapt existing workflows, and build trust in AI-generated recommendations. Furthermore, maintaining and updating these AI systems, including managing data privacy, applying software updates, and ensuring reliable Internet connectivity, requires resources and technical capabilities that may be limited, particularly in low-resource settings. Addressing these barriers through careful planning and infrastructure investment is essential for achieving meaningful real-world impact from AI tools in ASD care.

#### 4.2.9 Improving human-AI interactions

Another critical research priority involves optimizing interactions between autistic individuals and AI systems. Future studies should investigate which interaction modalities (e.g., text-based chatbots, voice assistants, robot embodiments) are most effective, comfortable, and accessible for ASD users. Research could explore methods for refining AI systems to better interpret ambiguous or minimal user inputs without requiring autistic individuals to adapt their communication style to technology. Additionally, user-centered prompt engineering specifically tailored to neurodivergent communication styles represents an important area of investigation (9, 50).

Studies such as the Pepper robot (49) suggest that tailoring human–robot interaction paradigms specifically to ASD users (e.g., employing simplified language, visual supports, or predictable robotic behaviors) can enhance usability and effectiveness. Collaborative research involving ASD specialists and user-experience experts can identify design principles most conducive to user engagement and therapeutic effectiveness. Enhancing human–AI interaction in these ways aims to strengthen user engagement, improve therapeutic alliance, and ultimately improve clinical outcomes for ASD individuals.

#### 4.2.10 Data augmentation and AI model development

Due to inherent limitations in ASD-related datasets, generative approaches that create synthetic data or augment existing samples play an increasingly important role. Such approaches include generating synthetic behavioral and neuroimaging data representative of autistic populations, simulating social environments or virtual individuals for AI model training, and applying transfer learning techniques to adapt general-purpose AI models to ASD-specific contexts (41). Evaluating the quality, bias, and practical utility of these generative data augmentation methods is critical, particularly in addressing challenges related to small sample sizes and dataset biases common in ASD research.

Additionally, future research directions include developing specialized AI models optimized for ASD-specific data. This involves designing neural network architectures and explainable AI frameworks specifically tailored to ASD datasets, thereby improving model performance, interpretability, and clinical relevance (10). Investigating the underlying infrastructure of AI, such as effective data augmentation strategies, feature generation techniques, and model refinement methodologies, will further advance ASD-focused AI applications (8). Thus, this research area encompasses not only the applied use of AI tools in practice but also the foundational methods by which AI systems for ASD diagnosis and intervention are developed, trained, and continually improved.

### 4.3 Limitations

We deliberately confined this scoping review to peer-reviewed literature to protect methodological reliability, even though many GenAI breakthroughs first surface as arXiv or medRxiv preprints. This choice inevitably omits some state-of-the-art approaches and may underestimate the current performance ceiling, but it preserves a minimum evidentiary standard across studies.

Within the included studies, research designs, model architectures, comparator choices, and outcome definitions remain highly heterogeneous. Although we translated disparate metrics into percentage-point changes or relative improvements for **Table 2**, the underlying variability still violates key assumptions for quantitative synthesis, leaving any meta-analytic aggregation or definitive cross-study ranking premature.

Evidence that is available tends to come from proof-of-concept pilots or tightly controlled laboratory experiments, often based on modest, demographically narrow samples. Such settings rarely reflect the complexity of real-world clinics, where comorbidities, environmental variability, and implementation logistics can dampen algorithmic performance. Compounding this limitation, nearly all evaluations report only immediate or short-term outcomes, so we cannot determine whether observed gains persist over months or translate into everyday functioning.

Finally, few articles provide transparent interpretability analyses, systematic bias audits, or detailed error-type breakdowns. These omissions hinder assessments of clinical safety, fairness, and trustworthiness—critical prerequisites for deploying GenAI tools with vulnerable populations such as autistic individuals. Collectively, these constraints indicate that the current evidence base is still preliminary and underscore the need for standardized outcome taxonomies, multi-site longitudinal trials with diverse cohorts, and built-in bias-mitigation and interpretability evaluations before GenAI systems can be considered ready for routine ASD assessment and intervention.

### 4.4 Conclusions

This scoping review highlights the growing promise of GenAI technologies in enhancing the assessment, intervention, and caregiver support for individuals with ASD. By synthesizing empirical studies across screening, therapeutic, and assistive domains, this review demonstrates that GenAI offers a flexible and scalable means to deliver personalized care. Early findings suggest improvements in diagnostic sensitivity, therapy engagement, and caregiver education. However, these benefits remain largely confined to proof-of-concept stages and have important limitations. Theoretically, current GenAI approaches lack interpretability and remain prone to hallucinations or confabulations, undermining trust in clinical decision-making. Standardized outcome metrics are also scarce, making cross-study comparisons difficult. Practically, most existing tools have only been tested on small, demographically narrow populations, often in lab-based environments. Few studies evaluate sustained impact, integration into clinical workflows, or the burden on caregivers and practitioners. These limitations underscore the need for rigorous validation, contextual adaptation, and inclusive deployment strategies to support the safe and effective adoption of GenAI systems

Future research should address key priorities to advance this field responsibly and effectively. These include: (1) developing architectures that integrate multimodal inputs such as speech, gaze, and movement; (2) enhancing transparency through XAI frameworks tailored to ASD-specific applications; (3) embedding ethics into the AI development process through participatory co-design with autistic individuals and caregivers; (4) rigorously testing GenAI tools in longitudinal, multi-site trials; and (5) addressing equity concerns by curating inclusive datasets and evaluating subgroup performance across gender, language, culture, and socioeconomic contexts.

From a policy perspective, the findings of this review have several implications. First, health agencies and regulatory bodies must begin formulating guidelines for the ethical deployment of GenAI in mental health and developmental care, including requirements for transparency, interpretability, and safety monitoring. Second, investment in digital infrastructure—particularly in low-resource and rural settings—will be essential to ensure equitable access to GenAI-enabled services. Third, training and certification standards for professionals working with GenAI-enhanced tools must be developed in collaboration with interdisciplinary experts. A coordinated policy response can help maximize the societal benefit of GenAI while safeguarding autistic individuals.

In summary, while GenAI presents exciting opportunities for advancing ASD care, realizing its full potential will require commitment to evidence-based design, ethical implementation, and inclusive policy support. Continued interdisciplinary collaboration among AI researchers, clinicians, ethicists, policymakers, and autistic communities will be key to ensuring that GenAI systems become reliable, equitable, and trusted components of future ASD services.

## Data Availability

The original contributions presented in the study are included in the article/**Supplementary Material**. Further inquiries can be directed to the corresponding author.
