# Supplementary material for: Implementation of generative AI for the assessment and treatment of autism spectrum disorders: a scoping review
Source: Front Psychiatry. 2025 Jul 22;16:1628216. doi: 10.3389/fpsyt.2025.1628216 (PMC12322814; doi:10.3389/fpsyt.2025.1628216)
Supplement: Supplementary file 1 [file DataSheet1.docx]

Supplementary Material

# Search String

## PubMed

("Artificial Intelligence"[MeSH Terms] OR "Artificial Intelligence"[All Fields] OR "Computational Intelligence"[All Fields] OR "Computer Reasoning"[All Fields] OR "Computer Vision Systems"[All Fields] OR "Knowledge Acquisition Computer"[All Fields] OR "Knowledge Representation Computer"[All Fields] OR "Machine Intelligence"[All Fields] OR "Deep Learning"[MeSH Terms] OR "Deep Learning"[All Fields] OR "Hierarchical Learning"[All Fields] OR "Machine Learning"[MeSH Terms] OR "Machine Learning"[All Fields] OR "Transfer Learning"[All Fields] OR "Neural Networks, Computer"[MeSH Terms] OR "Computational Neural Networks"[All Fields] OR "Connectionist Models"[All Fields] OR "Models Neural Network"[All Fields] OR "Neural Network Models"[All Fields] OR "Neural Networks computer"[All Fields])

## Embase

('childhood disintegrative disorder'/exp OR 'pervasive developmental disorder not otherwise specified'/exp OR 'rett syndrome'/exp OR

'autism'/exp OR 'autism spectrum disorder':ti,ab OR 'autistic spectrum disorder':ti,ab OR 'asperger syndrome'/exp OR 'asperger disease':ti,ab OR 'asperger disorder':ti,ab OR 'asperger syndrome':ti,ab)

AND

(('generative artificial intelligence':ti,ab OR 'generative ai':ti,ab OR 'generative model':ti,ab OR 'generative model*':ti,ab OR 'gpt':ti,ab OR 'generative pre trained transformer*':ti,ab OR 'gan':ti,ab OR 'generative adversarial network*':ti,ab OR 'llm':ti,ab OR 'large language model':ti,ab OR 'large language model*':ti,ab OR 'transformer*':ti,ab OR 'diffusion model*':ti,ab)

AND

('artificial intelligence'/exp OR 'artificial intelligence':ti,ab OR 'computational intelligence':ti,ab OR 'computer reasoning':ti,ab OR 'computer vision system':ti,ab OR 'knowledge acquisition computer':ti,ab OR 'knowledge representation computer':ti,ab OR 'machine intelligence':ti,ab OR 'deep learning'/exp OR 'deep learning':ti,ab OR 'hierarchical learning':ti,ab OR 'machine learning'/exp OR 'machine learning':ti,ab OR 'transfer learning':ti,ab OR 'artificial neural network'/exp OR 'computational neural network':ti,ab OR 'connectionist model':ti,ab OR 'models neural network':ti,ab OR 'neural network models':ti,ab OR 'neural networks computer':ti,ab))

## PsycINFO

TI,AB(( ("child development disorder*" OR "pervasive developmental disorder*" OR "rett syndrome" OR "autism spectrum disorder" OR "autistic spectrum disorder" OR "asperger syndrome" OR "asperger disease" OR "asperger disorder") ))

AND

TI,AB(( ("generative artificial intelligence" OR "generative AI" OR "generative model*" OR "GPT" OR "generative pre-trained transformer*" OR "GAN" OR "generative adversarial network*" OR "LLM" OR "large language model*" OR "transformer*" OR "diffusion model*") ))

AND

TI,AB(( ("artificial intelligence" OR "computational intelligence" OR "computer reasoning" OR "computer vision system*" OR "knowledge acquisition computer" OR "knowledge representation computer" OR "machine intelligence" OR "deep learning" OR "hierarchical learning" OR "machine learning" OR "transfer learning" OR "artificial neural network*" OR "computational neural network*" OR "connectionist model*" OR "neural network model*" OR "neural networks computer") ))

## Scopus

(TITLE-ABS-KEY("child development disorder*" OR "pervasive developmental disorder*" OR "rett syndrome" OR "autism spectrum disorder" OR "autistic spectrum disorder" OR "asperger syndrome" OR "asperger disease" OR "asperger disorder"))

AND

(TITLE-ABS-KEY("generative artificial intelligence" OR "generative AI" OR "generative model*" OR "GPT" OR "generative pre-trained transformer*" OR "GAN" OR "generative adversarial network*" OR "LLM" OR "large language model*" OR "transformer*" OR "diffusion model*"))

AND

(TITLE-ABS-KEY("artificial intelligence" OR "computational intelligence" OR "computer reasoning" OR "computer vision system*" OR "knowledge acquisition computer" OR "knowledge representation computer" OR "machine intelligence" OR "deep learning" OR "hierarchical learning" OR "machine learning" OR "transfer learning" OR "artificial neural network*" OR "computational neural network*" OR "connectionist model*" OR "neural network model*" OR "neural networks computer"))

## Web of Science

TS=("child development disorder*" OR "pervasive developmental disorder*" OR "rett syndrome" OR "autism spectrum disorder" OR "autistic spectrum disorder" OR "asperger syndrome" OR "asperger disease" OR "asperger disorder")

AND

TS=("generative artificial intelligence" OR "generative AI" OR "generative model*" OR "GPT" OR "generative pre-trained transformer*" OR "GAN" OR "generative adversarial network*" OR "LLM" OR "large language model*" OR "transformer*" OR "diffusion model*")

AND

TS=("artificial intelligence" OR "computational intelligence" OR "computer reasoning" OR "computer vision system*" OR "knowledge acquisition computer" OR "knowledge representation computer" OR "machine intelligence" OR "deep learning" OR "hierarchical learning" OR "machine learning" OR "transfer learning" OR "artificial neural network*" OR "computational neural network*" OR "connectionist model*" OR "neural network model*" OR "neural networks computer")
